# Draft Genome Sequence of the Host-Restricted *Salmonella enterica* Serovar Abortusovis Strain SS44

**DOI:** 10.1128/genomeA.00261-14

**Published:** 2014-04-03

**Authors:** M. Deligios, D. Bacciu, E. Deriu, G. Corti, R. Bordoni, G. De Bellis, G. S. Leori, S. Rubino, S. Uzzau

**Affiliations:** a Dipartimento di Scienze Biomediche, Università di Sassari, Sassari, Italy; b Consiglio Nazionale delle Ricerche, Istituto di Tecnologie Biomediche, Segrate, Italy; c Istituto Zooprofilattico Sperimentale della Sardegna, Sassari, Italy; d Department of Infection and Immunity, King Faisal Specialist Hospital and Research Centre, Riyadh, Saudi Arabia

## Abstract

*Salmonella enterica* serovar Abortusovis is a pathogen strictly adapted to ovines, in which it causes abortion. To enhance our understanding of this pathogen, we assembled the first draft sequence of an *S.* Abortusovis genome (strain SS44). The obtained genomic data might facilitate the study of *S. enterica* evolution and host adaptation.

## GENOME ANNOUNCEMENT

The ovine pathogen *Salmonella enterica* serovar Abortusovis is endemic in several European and Asian countries, where it causes significant economic losses due to the high rates of abortion in infected flocks. The introduction of *S.* Abortusovis epidemic strains in new areas is difficult to control due to the asymptomatic behaviors in infected adult lambs, rams, and nonpregnant ewes (1). Strain SS44 is a clinical isolate (2) that has been widely used to study *S.* Abortusovis virulence features by means of *in vitro* assays and animal experimental models (1, 3, 4). The genome sequence was obtained by a whole-genome shotgun strategy using the Roche 454 Life Sciences GS-20 sequencer and GS-FLX sequencer (478,585 reads totaling 51,883,128 bp). Reads from 3 pyrosequencing runs were assembled using the GS *de novo* Assembler software and GS Reference Mapper software (with published *Salmonella* genomes). Gaps were closed manually, reaching an assembly of 213 contigs (*N*_50_ length, 54,599 bp) corresponding to a total of 4,508,347 bp. The draft genome of SS44 has an estimated size of 4.6 Mb, with a G+C content of 52.1%. Contig annotation performed using the Prodigal version 1.20 analysis server (5) predicted 4,924 coding genes. Of these, 2,884 have a full-length homolog in other genomes of *S. enterica* strains (BLASTp protein identity, ≥80% for ≥80% protein length; *E* value, <10^-8^). Six contigs (42,800 bp in total) have a high similarity (>90%) with other *S. enterica* serovar virulence plasmids (GenBank accession no. AE006471, EU219534, AY517905, HE663166, and JN885080).

SS44 carries IS*1414*, a 1,344-bp insertion sequence (IS) that has been detected in several pathotypes of *Escherichia coli* infecting both humans and domesticated animals (3, 6). Read alignment with IS*1414*-borne *tnpA* gene sequence showed a coverage of ~100×, confirming a high copy number, as was previously suggested (3). As expected, the high number of IS*1414* copies affected the ability to automatically assemble the pyrosequencing reads and hampered the labor of gap closures through directed PCR and primer walking approaches. IS*1414* might have been recently transferred from *E. coli* into *S.* Abortusovis, since strains isolated in Asia do not carry this IS element (3). The adaptation of *S.* Abortusovis to sheep might also be recent, since high copy numbers of IS elements have been observed in the genomes of bacteria adapted only recently to a host-restricted pathogenic lifestyle (4, 7–10). Six *Salmonella* pathogenicity islands (SPI-1 to SPI-6) were reported to be present in SS44, with major degradation in SPI-3 and SPI-6 (of about 4 kbp and 20 kbp, respectively). Host-adapted and host-restricted serovars of *S. enterica* have been reported to depend on their environment for amino acids and essential nutrients (10). The SS44 draft genome shows that *S.* Abortusovis auxotrophy for cysteine and nicotinic acid is due to mutations in the *cysI*, *nadB*, and *nadC* genes. Further analysis of the SS44 genome will help to understand the *S.* Abortusovis evolution toward an ovine-restricted pathogenicity.

### Nucleotide sequence accession numbers.

This whole-genome shotgun project has been deposited at DDBJ/EMBL/GenBank under the accession no. AUYQ00000000. The version described in this paper is version AUYQ02000000.
